# Meeting of the East Midlands District Regional Committee

**Published:** 1920-11-13

**Authors:** 


### BRITISH HOSPITALS' ASSOCIATION.

### Meeting of the East Midlands District Regional Committee.

A meeting of twenty-eight representatives of sixteen voluntary hospitals was recently held at the General Hospital, Nottingham.

In the absence of Mr. F. Acton, the Chairman of the Nottingham General Hospital, Mr. James Forman, the Vice-Chairman of that hospital, presided.

Mr. H. Wade Deacon, Chairman of the Council of the British Hospitals' Association, addressed the meeting. He stated the Association was formed some years ago, and has proved itself a useful institution, but now there is need for better organisation to make it really representative of the hospitals. The governing body is the Council, which is elected annually, and the duties are carried on from a central office in London. The Association has been in touch for some years with the various Government Departments, including the Ministry of Pensions and the Ministry of Health. The Government wants a body to whom they can refer now and again when questions arise, which can say "We represent, broadly speaking, the views of the voluntary hospitals in the country." It had been decided, therefore, to form local branches of the Association, the basis to be the areas adopted by the Ministry of Pensions. The object of the branch will be to meet together from time to time as may be thought advisable or necessary. Members could discuss subjects of interest in common; no doubt valuable suggestions would be made, and the general welfare of the hospitals promoted. To strengthen the Council, or governing body of the Association, each branch will nominate a representative to sit on the Council, so that the latter will be in direct touch with the branches. In this way the Association will be better able to approach the Ministries from time to time, and answer questions, as the representative body of the voluntary hospitals of the country. There are many questions to be considered, such as, "What will be the relation of the voluntary hospitals to the State in future? Are we to be subsidised, taken over, or formed part of some general scheme formulated by the Ministry of Health? What arrangements can be made for increasing subscriptions and payments of patients?"

A discussion followed, and Mr. Harry Johnson, of the Leicester Royal Infirmary, severely criticised the work of the Association in the past. Mr. Wade Deacon answered several questions, and in reply to Mr. Johnson pointed out that the Association will be what the members like to make it.

The following resolution was moved by Admiral Sir Frederick Inglefield: "Resolved that this meeting welcomes the proposal to establish a branch of the British Hospitals' Association, representing the voluntary hospitals in Derbyshire, Nottinghamshire, Lincolnshire, Leicestershire, Northamptonshire, and the county of Rutland, and undertakes to support such a branch Association."

Mr. E. C. Barnes, of the North Derbyshire Hospital, Chesterfield, seconded the resolution, which was carried unanimously.

The appointment of a chairman, vice-chairman, and a representative to serve on the Council was postponed to a future meeting.

Mr. Walter Banks, of the Derbyshire Royal Infirmary, was selected as the honorary secretary, and was given authority to call the next meeting.

